# Advancing the Landscape of Clinical Actionability in Von Hippel–Lindau Syndrome: An Evidence-Based Framework from the INT^2^GRATE Oncology Consortium

**DOI:** 10.3390/cancers17132173

**Published:** 2025-06-27

**Authors:** Diane R. Koeller, McKenzie Walker, Busra Unal, Anu Chittenden, Alison Schwartz Levine, Connor P. Hayes, Paul C. Oramasionwu, Monica D. Manam, Ryan M. Buehler, Israel Gomy, Wilson Araujo Silva, Jordan Lerner-Ellis, Selina Casalino, Radhika Mahajan, Nicholas Watkins, Nihat Bugra Agaoglu, Danielle K. Manning, Justine A. Barletta, Jason L. Hornick, Neal I. Lindeman, Lynette M. Sholl, Huma Q. Rana, Judy E. Garber, Arezou A. Ghazani

**Affiliations:** 1 Division of Cancer Genetics and Prevention, Dana-Farber Cancer Institute, Boston, MA 02215, USA; 2 Division of Genetics, Brigham and Women’s Hospital, Boston, MA 02115, USA; 3 Boston Children’s Hospital, Boston, MA 02115, USA; 4 Department of Genetics, Ribeirão Preto Medical School, University of São Paulo, Ribeirão Preto 14040-902, Brazil; 5 Pathology and Laboratory Medicine, Mount Sinai Hospital, Toronto, ON M5G 1X5, Canada; 6 Division of Cancer Genetics, Umraniye Training and Research Hospital, 34764 Istanbul, Turkey; 7 Department of Pathology, Brigham and Women’s Hospital, Boston, MA 02115, USA; 8 Harvard Medical School, Boston, MA 02114, USA; 9 Pathology and Laboratory Medicine, Weill Cornell Medical College, New York, NY 10065, USA; 10 Division of Population Sciences, Dana-Farber Cancer Institute, Boston, MA 02115, USA; 11 Department of Medicine, Brigham and Women’s Hospital, Boston, MA 02115, USA

**Keywords:** INT^2^GRATE Oncology Consortium, somatic and germline integration, Von Hippel–Lindau Syndrome, INT^2^GRATE variant portal, VHL

## Abstract

**Background/Objectives:** An accurate evaluation of variant actionability is essential in cancer management. In Von Hippel–Lindau Syndrome (VHL), the interpretation of the germline variants is confounded by the presence of non-syndromic component tumors, such as clear cell renal cell carcinoma (ccRCC), hemangioblastoma, pheochromocytoma, and neuroendocrine tumors. These tumors frequently occur sporadically, without any association with VHL syndrome. The presence of these tumors in a patient with a germline *VHL* variant could lead to inaccurate attribution of these tumors to the germline variant and VHL syndrome. In our previous INT^2^GRATE (INTegrated INTerpretation of GeRmline And Tumor gEnomes) programs, we demonstrated that integrating tumor-derived and germline evidence offers a comprehensive approach for the accurate assessment of the germline variants in cancer syndromes. **Methods/Results:** Here, we present a novel INT^2^GRATE variant evidence framework (VEF) for evaluating the clinical actionability of the germline variants in VHL syndrome, offering an integrated approach that incorporates both constitutional and tumor data. We analyzed 2672 variants in the *VHL* gene and their associated tumors and clinical evidence to effectively distinguish between constitutional, sporadic, VHL differentials, and *VHL* allelic genetic conditions. The germline INT^2^GRATE variants, along with their comprehensive associated evidence, were made accessible in the first open-access INT^2^GRATE Variant data Portal. **Conclusions:** This novel and integrated approach to variant assessment and data sharing in hereditary cancer syndromes is essential in the clinical evaluation of genomic variants, advancing precision oncology, and improving patient care.

## Simple Summary

In cancer syndromes, a precise assessment of variant actionability requires a comprehensive set of evidence to delineate the role of a variant in disease while effectively distinguishing it from potential differential diagnoses with similar cancer presentations and from sporadic occurrences. The INT^2^GRATE programs and the INT^2^GRATE Oncology Consortium address this challenge through an integrated analysis of constitutional and tumor data. Here, we present a novel variant evidence framework (VEF) for precision variant assessment in Von Hippel–Lindau Syndrome (VHL). The INT^2^GRATE VEF, applied to 2672 *VHL* variants, distinguishes constitutional, sporadic, VHL differential, and *VHL* allelic conditions. We also present the open-access INT^2^GRATE Variant Portal, a novel resource that provides a repository of the germline *VHL* variants and evidence, promoting data sharing, advancing precision oncology, and improving patient care.

## 1. Introduction

Von Hippel–Lindau syndrome (VHL, OMIM # 193300) is a rare, autosomal dominant hereditary cancer syndrome caused by the heterozygous germline pathogenic variants in the *VHL* gene. With an estimated prevalence of 1 in 35,000 [1], VHL syndrome is characterized by the presence of multiple tumor types, specifically clear cell renal cell carcinoma (ccRCC), hemangioblastomas of the central nervous system and retina, pheochromocytomas, pancreatic cysts, and neuroendocrine tumors [2,3]. The mechanism of disease in VHL syndrome is the loss of function of the *VHL* tumor suppressor gene, leading to the accumulation of hypoxia-inducible factors (HIFs) and the formation of highly vascularized tumors [4,5,6]. Nearly 100% of the individuals with VHL syndrome are expected to be symptomatic by the age of 65 years, but some germline *VHL* variants may be associated with lower penetrance [1,7,8].

**Figure 1:**
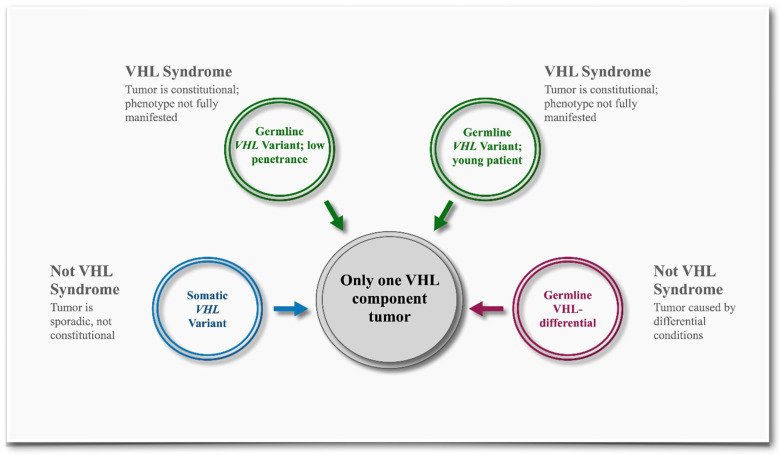
Possible genetic etiologies of an isolated VHL tumor. The diagram depicts the molecular mechanisms underlying the development of a VHL component tumor. An isolated VHL tumor, such as renal cell carcinoma, may be sporadic and not related to VHL syndrome. It may be related to differential genetic conditions with clinical features resembling those in VHL syndrome. Alternatively, a VHL component tumor can be related to a germline *VHL* variant with incomplete penetrance, age-related penetrance, or high penetrance but in a young patient.

The interpretation of the germline *VHL* variants and the assessment of their clinical significance in VHL syndrome can be complex. Often, patients suspected of having VHL syndrome present with a single VHL component tumor, such as isolated RCC, hemangioblastoma, pheochromocytoma, or retinal angioma. Non-syndromic component tumors may be common sporadic occurrences that do not involve germline *VHL* alterations. These sporadic tumors have distinct tumorigenic drivers, often involving somatic alterations in the *VHL* or other genes. The allelic autosomal recessive disorder familial erythrocytosis type 2 (ECYT2, OMIM # 263400) adds further complexity, as the *VHL* germline variants related to ECYT2 are generally not associated with VHL syndrome. Alternatively, component tumors may be constitutional, caused by germline alterations in genes other than the *VHL*. Genes such as *FH*, *FLCN*, *MET*, *BAP1*, *SDHA*, *SDHB*, *SDHC*, *SDHD*, *SDHAF2*, *TMEM127*, *MAX*, and *RET* are associated with cancer susceptibility syndromes that present overlapping tumors commonly seen in VHL syndrome [9]. Finally, a single VHL-component tumor could be attributed to age-related penetrance or the low penetrance of certain *VHL* alleles in adults (Figure 1). These observations highlight the confounding factors in the clinical interpretation of the *VHL* variants and underscore the need for an integrated and comprehensive evidence-based assessment of the *VHL* variants.

The INT^2^GRATE|Oncology Consortium has addressed this challenge by developing an integrated evidence framework to assess the actionability of the germline variants using germline data, clinical genetic information, tumor-derived data, and somatic genetic evidence (INT^2^GRATEoncology.org). We have previously presented the application of INT^2^GRATE in the assessment of variant actionability in multiple hereditary cancer syndromes, and further demonstrated that this comprehensive approach can differentiate between non-syndromic sporadic tumors or syndromic cancers [8,10,11,12,13]. Here, we present the novel INT^2^GRATE variant evidence framework (VEF) for evaluating the germline variants in VHL syndrome. We describe the parameters and rationale behind the VEF, discuss the collation and programmatic processing of 2672 variants in the INT^2^GRATE|VHL program, and unveil the launch of the first open INT^2^GRATE|Variant Portal for public access. This novel framework integrates evidence derived from constitutional and sporadic variants, VHL differential conditions, and allelic conditions. Processing the INT^2^GRATE|Variants and sharing comprehensive constitutional and somatic evidence is pivotal in advancing precision oncology.

## 2. Methods

### 2.1. Development of the INT^2^GRATE|VHL Platform

#### INT^2^GRATE|VHL Variant Evidence Framework (VEF)

**Figure 2:**
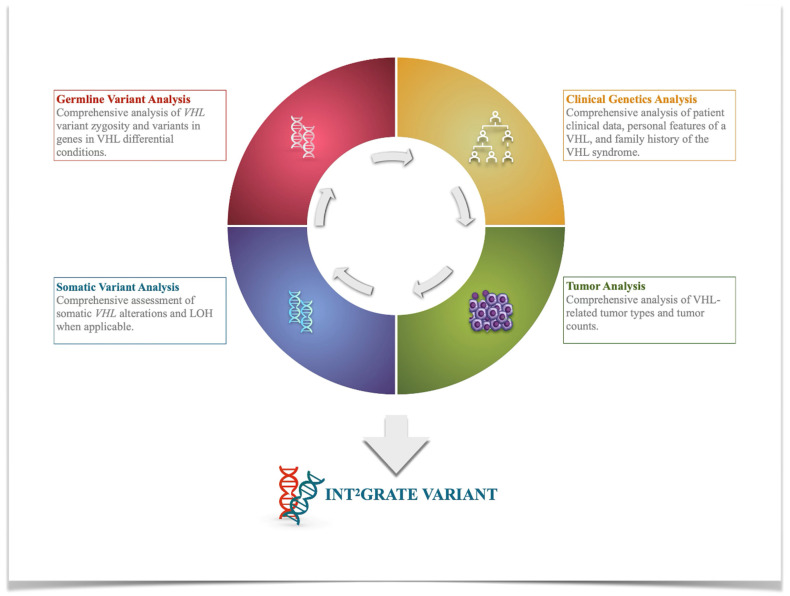
Schematic representation of the INT^2^GRATE|VHL. The INT^2^GRATE variant evidence framework (VEF) has the following four main components: (1) germline variants, including the *VHL* variants and the zygosity status, and variants in genes related to VHL differential conditions; (2) pertinent patient clinical genetics data related to personal and family history of VHL syndrome; (3) tumor-derived data, including VHL-related tumor counts and types; and (4) somatic genetic variants in the *VHL* gene. A comprehensive evaluation of the INT^2^GRATE variants can help differentiate their role in non-syndromic sporadic tumors or syndromic VHL, *VHL* allelic disorders, and differential conditions.

The variant evidence framework (VEF) for the INT^2^GRATE|VHL has four main components, including (1) germline variants in the *VHL* gene, (2) pertinent patient clinical genetics data related to personal and family history of VHL syndrome, (3) VHL tumor-derived data, and (4) somatic genetic variants in the *VHL* gene (Figure 2). The VEF was established by cataloging different clinical scenarios, marking the presence or absence of each variant parameter, and assessing the combination of the evidence in each scenario (Supplementary Materials Table S1). The details of the VEF and clinical scenarios were reviewed by the INT^2^GRATE expert group, comprising board-certified medical geneticists with experience in cancer genetics and tumor profiling, board-certified clinicians, and genetic counselors with expertise in the diagnosis and management of hereditary cancer syndromes, including VHL, and board-certified pathologists with expertise in tumor pathology. The description and rationale for the VEF components are described in detail in the Results (Section 3).

### 2.2. INT^2^GRATE Data Processing and Analysis

#### 2.2.1. Development of the Digital INT^2^GRATE VEF

Once the INT^2^GRATE expert committee signed off on the VEF, a digital version was developed at the INT^2^GRATE Data Coordinating Center to support large-scale data analysis. The digital VEF was developed in Python (https://www.python.org/psf-landing/ Python Software Foundation, Wilmington, DE, USA) through a series of logical combinations for each scenario.

#### 2.2.2. INT^2^GRATE Encoder

The INT^2^GRATE Encoder was developed in Python to pragmatically analyze the data provided by the INT^2^GRATE institute members by parsing them through the digital VEF. The Encoder integrates all VEF parameters in each case, identifies the matching INT^2^GRATE scenario, and assigns the case to the relevant unique INT^2^GRATE code. In the resulting output file, all variants were labeled with the appropriate unique INT^2^GRATE code as determined by the VEF. Each patient was assigned a unique Subject ID.

#### 2.2.3. INT^2^GRATE Pattern Quantifier

The INT^2^GRATE Pattern Quantifier was developed in Python to assess, identify, and quantify unique patterns among the *VHL* variants in the dataset. First, it computed the frequency of each variant, which was termed variant recurrence. For each variant, it further assessed and quantified the distinct patterns of the supporting evidence (e.g., INT^2^GRATE Evidence 1, INT^2^GRATE Evidence 2). Then, it enumerated the number of times that an identical pattern of evidence was observed for a given variant (e.g., *n* = 3). The INT^2^GRATE Pattern Quantifier facilitates a large-scale investigation of the landscape and the distribution of variants, along with their associated clinical evidence.

#### 2.2.4. INT^2^GRATE|Data Portal and Data Sharing

The INT^2^GRATE Data Portal is a novel digital platform for sharing the de-identified INT^2^GRATE variants and their associated and comprehensive clinical evidence. After formatting the variants through the INT^2^GRATE Encoder and Pattern Quantifier, the aggregate dataset was uploaded to the portal through a web-based user interface available at INT^2^GRATEdata.bwh.harvard.edu.

### 2.3. Patient Data Query and Cohorts

In total, data from 2672 patients were retrospectively collected and analyzed in the INT^2^GRATE VHL program. An agnostic approach included all *VHL* variant-positive cases (at least one germline or somatic *VHL* variant) without prior knowledge of VHL syndrome evaluation to minimize ascertainment bias. The INT^2^GRATE VEF was provided to the consortium members to ensure a uniform process for querying relevant data. Member institutes include Mass General Brigham (MGB, Boston, MA, USA), Dana Farber Cancer Institute Center for Cancer Genetics and Prevention (DFCI, Boston, MA, USA), Mount Sinai Hospital (MSH, Toronto, ON, Canada), University of São Paulo (USP, Ribeirão Preto, São Paulo, Brazil), and Umraniye Training and Research Hospital (UTRH, Istanbul, Turkey).

Data were divided into two cohorts. Cohort 1 consisted of 133 patients, 122 of whom had a germline *VHL* variant and detailed tumor data and were evaluated at a genetics clinic. The remaining 11 patients all had the same data plus somatic *VHL* genetic information. Cohort 2 included 2539 patients, all of whom had at least one somatic *VHL* variant and detailed tumor data. A subset of these patients (*n* = 638) was evaluated in the genetics clinic and had no germline *VHL* variants, while the remaining 1901 patients had no germline testing and no record of clinical genetic evaluation for any constitutional cancer. About 94% of the somatic alterations were copy numbers (154 SNVs and 2394 copy numbers that included 1817 deletions). In the absence of germline sequencing data, it was unclear whether the *VHL* alterations identified through tumor sequencing were true somatic events. However, given that germline *VHL* deletions occur in only ~10% of VHL syndrome cases, and these cases did not have VHL-associated tumors, the alterations identified through tumor sequencing were presumed to be somatic events. In cases with VHL-related component tumors (RCC, hemangioblastoma, paraganglioma, and pancreatic neuroendocrine tumor), the *VHL* alterations from tumor sequencing were extrapolated to be presumed somatic due to the absence of clinical suspicion for VHL syndrome and the variant allelic fractions (VAF) outside of the expected heterozygous range (median VAF < 50 = 27%; median VAF > 50 = 68%). Nonetheless, because germline sequencing was not performed for these patients, these variants were only used for landscape analysis.

For germline variant collection, each institution collected all types of *VHL* variants, including SNVs, indels, or copy numbers (CNs) reported as pathogenic, likely pathogenic, VUS, or favor polymorphism in their routine clinical care. Favor polymorphism classification was assigned by the reporting laboratory to variants that were downgraded from VUS after their initial report. These variants were entered as benign/likely benign (B/LB) in this study. Patients with the germline *VHL* variants were also investigated for germline findings in the genes associated with syndromes that are in the differential diagnosis for VHL syndrome. These genes included *MAX*, *SDHA*, *SDHAF2*, *SDHB*, *SDHC*, *SDHD*, *TMEM127*, *NF1*, *RET*, *FH*, and *FLCN*. Comprehensive clinical information was collected and evaluated, which included personal and familial cancer history, the component tumor types, sex, and age at diagnosis.

### 2.4. Germline Genetic Laboratory Testing

Germline genetic testing was performed at multiple sites, including MSH, UTRH, USP, or commercial laboratories (Invitae Corp., San Francisco, CA, USA; Ambry Genetics, Aliso Viejo, CA, USA). Briefly, DNA was extracted from peripheral blood, and targeted regions were enriched by a hybridization-based protocol according to standard protocols [10]. Sequencing was carried out on a next-generation sequencing (NGS) platform. In the exonic regions, copy number variations were assessed by analyzing each target sequence read depth, along with the mean read depth and distribution of the read depth, based on the parameters established in the validation experiments.

### 2.5. Tumor Genetic Laboratory Testing

Somatic variants were obtained from routine tumor profiling by the OncoPanel assay at the Center for Advanced Molecular Diagnostics (CAMD) at BWH (Boston, MA, USA). Data were retrospectively collected through a custom search for all cases between 2018 and 2022 with the reported *VHL* variants as previously described [10]. Briefly, single nucleotide variants, copy numbers, and structural alterations were processed as below. Somatic loss of heterozygosity (LOH) was assessed by detecting allelic loss at the *VHL* locus.

#### 2.5.1. Single-Nucleotide Variant (SNV)/Indel Analysis

Per the previously described protocols, MuTect (Broad Institute, Cambridge, MA, USA) and GATK Indelocator (Broad Institute, Cambridge, MA, USA) were used to detect somatic SNVs and indels, respectively. The variant filtration was performed based on the annotated allele frequencies in the Exome Sequencing Project (ESP) and/or gnomAD, excluding the variants with an allelic frequency higher than 0.1% in these databases. The variants that were found in the plate normal control were excluded from further analysis. Any variant filtered based on the previous criteria but reported in the COSMIC database (COSMIC, Wellcome Sanger, London, UK) at least two times was rescued. The following information was used to annotate each variant: the gene, genome coordinates, reference and alternate alleles, coverage, allele fraction, cDNA, and protein change. According to the somatic OncoPanel validation data, 50× coverage and 10% variant allele fraction were set as the limit of detection. The variants failing these quality criteria for coverage and/or allele fraction or read support (at least five unique reads) were not further analyzed.

#### 2.5.2. OncoPanel Copy Number Analysis

As previously described, the somatic copy number (CN) variants were identified using RobustCNV, a tool developed at the Dana–Farber Cancer Institute (DFCI, Boston, MA, USA). The normalization of each baited genomic segment was performed by comparing it with a panel of normal samples. Genomic segments with a Log2 ratio of zero were classified as having a neutral copy number. Prior to the technical evaluation, copy number variations, including low and high amplifications, as well as one- and two-copy deletions, were recorded. A Log2 ratio of 0.43 or higher was used to identify low-level amplifications, and a Log2 ratio of −0.32 or lower indicated a copy number loss.

#### 2.5.3. OncoPanel Structural Variant Analysis

BreaKmer, created at DFCI (DFCI, Boston, MA, USA), was utilized in the assessment of somatic structural variants, including chromosomal rearrangements, inversions, and large indels, as previously described [14]. BreaKmer detects sequence segments that align to non-contiguous regions of the reference. For each structural variant (SV), the involved gene(s), genomic coordinates, and a corresponding IGV snapshot were provided for visual confirmation. The pipeline identifies split reads, single reads that align to two non-contiguous locations of the genome, and discordant read pairs in which the paired ends map to different locations in the genome. Variants with equal or less than 2% of total split and discordant reads/total coverage along the detected breakpoints were carefully assessed. SVs in repetitive regions were not included in the analysis.

## 3. Results

### 3.1. Development of INT^2^GRATE|Variant Evidence Framework (VEF) for VHL

The INT^2^GRATE|VEF incorporates four key types of evidence to assess the clinical relevance of the *VHL* variants (Figure 2), including germline genetics, somatic genetics, clinical genetics, and tumor-derived data. The INT^2^GRATE|VEF is designed to analyze the details within each category in different realistic scenarios routinely used in clinical practice. The details and rationale for each type of evidence are described below.

#### 3.1.1. Germline Variants and Rationale

The INT^2^GRATE|VEF requires a comprehensive assessment of the *VHL* zygosity, allelic disorder, and differential conditions to ensure a conservative evaluation of the germline variant associated with VHL syndrome.

*VHL* allelic disorder: While deleterious heterozygous *VHL* variants can be diagnostic for VHL syndrome, certain variants in the *VHL* gene are associated only with an increased risk for autosomal recessive familial erythrocytosis type 2 (ECYT 2) (also known as *VHL*-associated polycythemia, congenital erythrocytosis type 2, or Chuvash polycythemia). Individuals who are heterozygous for these variants are considered to be carriers of ECYT2 and are not expected to be at risk for VHL syndrome. Additionally, if an individual had bi-allelic *VHL* variants, it would be challenging to assess each variant separately in relation to any associated phenotypic features. Thus, zygosity is the first criterion in the assessment of the *VHL* variants, with the INT^2^GRATE|VEF for VHL only considering monoallelic (i.e., heterozygous) variants.

VHL differential disorders: Alterations in several genes can result in genetic conditions with clinical features resembling those in VHL syndrome. To accurately assess the potential association between a germline *VHL* variant and VHL syndrome, the INT^2^GRATE|VEF requires the absence of germline variants in genes related to differential disorders (Supplementary Materials Table S2). This conservative approach minimizes any potential confounding factors when evaluating the actionability of germline variants, especially in cases with unusual disease presentations where only non-syndromic tumors are present. The genes related to VHL differential disorders include *FH*, *FLCN*, *MET*, *BAP1*, *SDHA*, *SDHB*, *SDHC*, *SDHD*, *SDHAF2*, *TMEM127*, *MAX*, *RET*, and *NF1*, which are collectively associated with an increased risk of pheochromocytoma, paraganglioma, and renal cell carcinoma (RCC) including ccRCC. Table S2 in the Supplementary Materials lists each gene along with its associated VHL-differential condition(s).

#### 3.1.2. Clinical Genetics Criteria and Rationale

In the design of the INT^2^GRATE|VEF, the parameters for personal and family history of VHL are intentionally structured with different criteria. Personal history has minimal requirements, while family history has stringent criteria. This approach ensures a broader inclusion of cases while maintaining a more conservative analysis.

Personal history: While the presence of two or more VHL tumors is strongly suggestive of VHL syndrome, isolated VHL component tumors [15,16] (e.g., clear cell renal cell carcinoma, hemangioblastoma) can be either constitutional or sporadic. The VEF requires at least one VHL-related tumor or feature in the personal history. This low threshold allows for the inclusion and assessment of a broader range of cases with various tumor types (Supplementary Materials Table S1). The VEF captures the following VHL-related tumors: retinal hemangioblastoma, CNS hemangioblastoma, renal cell carcinoma, pheochromocytoma, pancreatic neuroendocrine tumor, endolymphatic sac tumor, and paraganglioma. VHL-related features include multiple kidney cysts, multiple pancreatic cysts, and epididymal and broad ligament cysts.

Family history: The family history requirement is designed to be very conservative. To meet the VEF criteria, family history is considered when a first or second-degree family member is diagnosed with VHL syndrome.

#### 3.1.3. Tumor-Derived Information and Rationale

A key feature of the INT^2^GRATE|VEF is its ability to distinguish between the various molecular etiologies of VHL tumors. An isolated VHL tumor may be (i) sporadic, where somatic *VHL* variants are putative drivers and the tumor is not related to VHL syndrome, (ii) caused by a germline variant in a gene related to VHL differential conditions, (iii) the result of a *VHL* germline variant in a young patient, or (iv) a VHL variant with low penetrance, which may result in an incomplete VHL phenotype (Figure 1). The INT^2^GRATE|VEF evaluates VHL tumor characteristics in the context of the patient’s clinical details to determine the potential tumor etiology and the association of the germline variant with VHL syndrome.

##### Only One VHL Component Tumor: Renal Cell Carcinoma

RCC, particularly clear cell histology, is common in VHL syndrome, occurring in 70% of the affected individuals. However, only 5–8% of RCCs are expected to be hereditary [17]. Because of the common occurrence of sporadic RCC, the VEF requires somatic genetic evidence to ensure an accurate evaluation of the germline *VHL* variants.

##### Only One VHL Component Tumor: Except Renal Cell Carcinoma

Non-RCC tumors are common in VHL syndrome, though they occur less frequently in the general population compared to RCC. With the exception of retinal hemangioblastomas, most non-RCC tumors associated with the *VHL* are sporadic. Therefore, when only one tumor other than RCC is present, the VEF requires additional evidence to support an association between a *VHL* variant and VHL syndrome.

##### Two or More VHL Component Tumors

Multiple VHL-related tumors can be highly suggestive of VHL syndrome, but the type of tumor (RCC vs non-RCC) impacts the assessment. Due to the common occurrence of sporadic RCC, including bilateral RCC tumors, the INT^2^GRATE|VEF considers scenarios in this category where one, two, or no RCC tumors are present (Supplementary Materials Table S1, Codes I–I, I–II, I–IV, II–I, II–IV, II–VII scenarios). The presence of multiple non-RCC VHL-associated tumors is more suggestive of VHL syndrome.

##### Non-VHL Tumor(s)

The INT^2^GRATE|VEF captures scenarios where the patient has neither VHL tumors nor any tumors at all. The absence of a VHL tumor, combined with the absence of relevant VHL-specific evidence, suggests that the germline *VHL* variant is not associated with VHL syndrome.

#### 3.1.4. Somatic Variants and Rationale

The INT^2^GRATE|VEF can distinguish between sporadic VHL tumors and those arising constitutionally as part of VHL syndrome. When a tumor is suspected to be sporadic, the INT^2^GRATE|VEF requires additional steps to assess somatic *VHL* alleles. Biallelic somatic *VHL* inactivating variants may indicate sporadic tumors, while a monoallelic somatic inactivating variant could represent the second hit in a constitutional tumor, where the first hit is the germline *VHL* variant.

### 3.2. Assignment of INT^2^GRATE Categories

INT^2^GRATE evaluates the combination of evidence across different scenarios to determine the relationship between the *VHL* variant and VHL disease. Each scenario is characterized by an INT^2^GRATE code along with a detailed comment that explains the reasoning behind the association (or absence of association) between the germline variants and VHL disease (Supplementary Materials Table S1).

INT^2^GRATE Positive designates scenarios in which all evidence parameters align conservatively with VHL syndrome (Supplementary Materials Table S1, Codes I–I to I–IV). Conversely, INT^2^GRATE Negative refers to cases where the evidence pattern does not support VHL syndrome (Supplementary Materials Table S1, Codes III–I to III–III). INT^2^GRATE Neutral represents scenarios where the evidence is strong, but not sufficient for a complete VHL variant assessment (Supplementary Materials Table S1, Codes II–I to II–VII). Lastly, INT^2^GRATE NOS denotes cases where a key piece of evidence is absent that prevents any assessment (Supplementary Materials Table S1, Codes IV–I to IV–III).

### 3.3. Patient Cohorts and Clinical Presentations

An agnostic approach was used in this study to select patients based on their *VHL* variant status rather than their personal history of VHL syndrome, thereby reducing potential ascertainment bias. Data from 2672 patients were collected from five institutions and analyzed at the INT^2^GRATE Coordinating Center (Table 1). Data were categorized based on the availability of germline sequencing data, clinical genetics evaluation, tumor details, and somatic sequencing data.

The INT^2^GRATE variant analysis was performed on the germline *VHL* variants from 133 patients evaluated in a genetics clinic for cancer predisposition, all of whom had tumor data, with a subset also having somatic sequencing data (Cohort 1; Table 1). Cohort 1 comprised 98 females (74%) and 35 males (26%), with the median age of cancer diagnosis at 54 years old (Table 2) presented with various VHL tumors, including hemangioblastomas and renal lesions (Table 3, Supplementary Materials Table S3).

A tumor landscape analysis was conducted on the remaining 2539 patients who were positive for the somatic *VHL* variant(s), had comprehensive tumor data, and a subset of whom had been evaluated in genetics but did not have the germline *VHL* variants (Cohort 2; Table 1).

#### 3.3.1. VHL Personal and Family History

**Figure 3:**
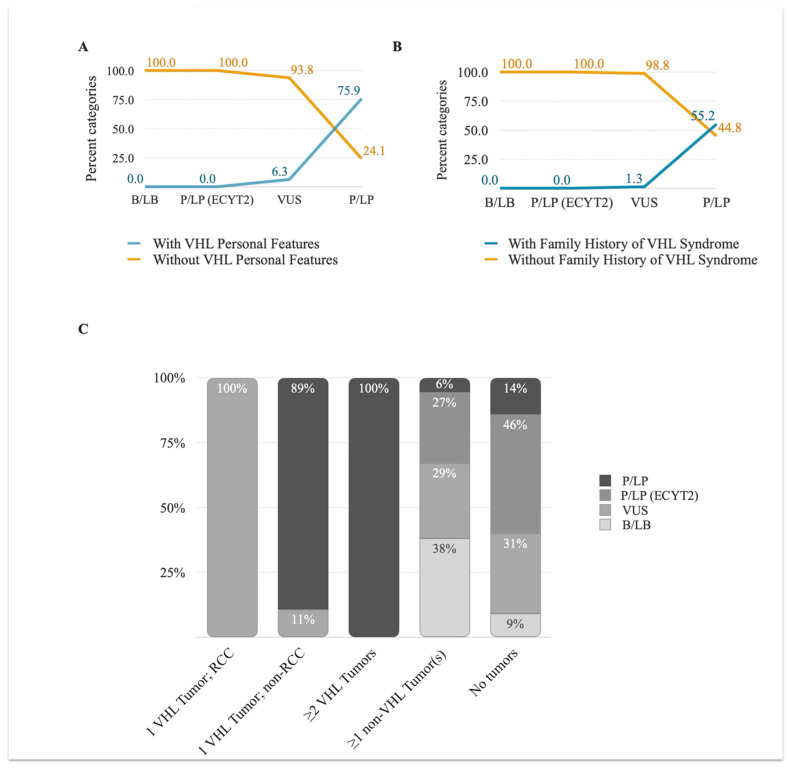
The clinical history and VHL tumors across patient cohorts before the INT^2^GRATE analysis. The (**A**,**B**) panels show the proportion of patients with VHL personal features and/or tumors (**A**) and family history of VHL syndrome (**B**). The variant classifications (ACMG) provided by the reporting laboratory are included on the X-axis. Panel (**C**) shows the distribution of VHL tumors across the variants according to their ACMG classifications. P/LP: pathogenic/likely pathogenic variants; P/LP (ECYT2): pathogenic/likely pathogenic variants related to ECYT2; VUS: variants of uncertain significance; B/LB: benign and likely benign variants.

Prior to performing the INT^2^GRATE analysis, the patients’ clinical data and the germline variant pathogenicity (as reported by the clinical laboratory) from Cohort 1 were evaluated. Among the germline *VHL* variants from 133 patients, 60% (80/133) were VUS. A small proportion of patients with germline VUS showed personal features and/or tumors and a family history of VHL diagnosis (6.3% and 1.3%, respectively) (Figure 3A). Among the 29 patients with pathogenic/likely pathogenic *VHL* variants, 26% did not exhibit VHL personal features and/or tumors, and 45% were negative for a family history of VHL syndrome (Figure 3B). In the remaining patients, none with polymorphic benign variants (*n* = 15) or monoallelic pathogenic variants associated with familial erythrocytosis (*n* = 9) exhibited personal features and/or tumors or a family history of VHL syndrome.

#### 3.3.2. VHL Tumor Information

The distribution of VHL component tumors among the patients in this study displayed a broad spectrum (Figure 3C). Among the patients with P/LP variants, only 65.5% (19/29) had ≥2 VHL component tumors, 10% (3/29) had only one component tumor, and another 14% (4/29) had only non-VHL tumors. The remaining 10% (3/29) had no tumors. The difference was even wider among the patients with VUS variants: 71% (57/80) had only non-VHL component tumors, 23% (18/80) had no tumors, and 5% (4/80) and 1% (1/80) had isolated RCC and isolated paraganglioma, respectively. All patients with B/LB or P/LP-ECYT2 monoallelic variants either had no VHL tumors or no tumors at all. The broad distribution of the *VHL* variants in the context of personal and family history or VHL tumor status highlights the inefficiency of assessing the evidence in isolation and the need for an integrated evaluation of evidence using INT^2^GRATE.

### 3.4. INT^2^GRATE|VHL Variant Analysis

**Figure 4:**
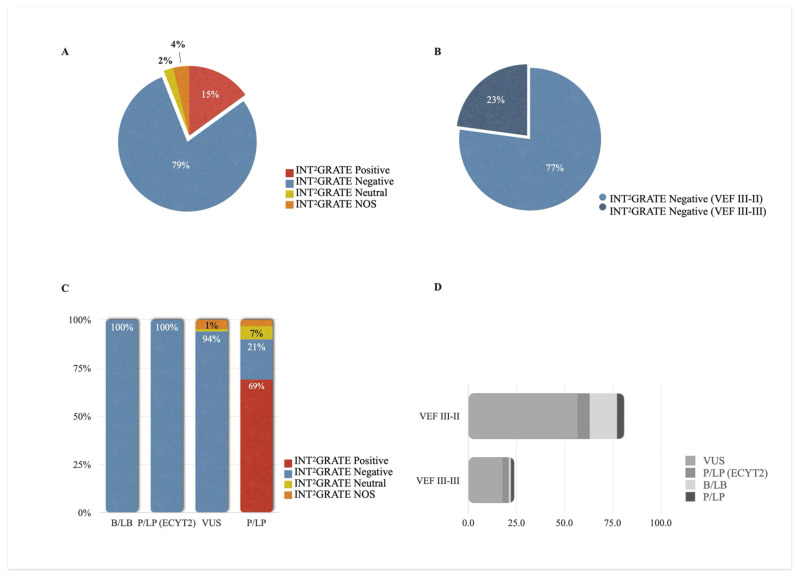
Distribution of Informative INT^2^GRATE Variants in the *VHL*. An INT^2^GRATE analysis resulted in 94% of germline VHL variants having informative INT^2^GRATE categories. This includes 15% INT^2^GRATE Positive and 79% INT^2^GRATE Negative variants (**A**). The INT^2^GRATE Negative category does not support the involvement of the germline variants in VHL syndrome; 77% of these variants were in adult patients with tumors other than VHL tumors (Code III–II), and 23% were in patients with no tumors (III–III) (**B**). The distribution of variants with different INT^2^GRATE categories and their reported ACMG classifications are shown in (**C**), and the breakdown of INT^2^GRATE Negative III–II and III–III is shown in (**D**). INT^2^GRATE Neutral represents the variants with strong but insufficient evidence for a complete evaluation, and INT^2^GRATE NOS denotes cases where key evidence was absent, excluding them for evaluation.

The INT^2^GRATE|VHL analysis was performed on 133 germline *VHL* variants. An input file containing all detailed data points was processed programmatically through the digital VEF to generate the INT^2^GRATE variants and associated codes. Overall, 94% of variants had informative INT^2^GRATE categories. This includes 79% (105/133) INT^2^GRATE Negative variants and 15% (20/133) INT^2^GRATE Positive variants. The assessment of the remaining 6% did not provide an informative status (2% INT^2^GRATE Neutral and 4% INT^2^GRATE NOS) (Figure 4A).

Consistent with a conservative VEF, all patients with the INT^2^GRATE Positive variants (*n* = 20) exhibited classic VHL syndrome (Supplementary Materials Table S4, Online INT^2^GRATE Portal). A total of 105 patients had the INT^2^GRATE Negative variants (Figure 4A), suggesting that the integrated evidence does not support the involvement of these germline variants in VHL syndrome in these patients. In this category, 77% (81/105) of the variants met the criteria for INT^2^GRATE III–II, while 23% (24/105) were INT^2^GRATE III–III, consistent with the absence of VHL tumors or any other tumors (Figure 4B, Supplementary Materials Tables S3–S6, Online INT^2^GRATE Portal).

Three patients had the INT^2^GRATE Neutral variants (i.e., insufficient for full analysis; Supplementary Materials Table S7). Patient 21 was positive for *VHL*:c.208G>A with no personal features or tumors of VHL but a first-degree relative diagnosed with VHL syndrome, meeting the criteria for II–VI. This scenario indicates that the absence of a personal phenotype might be related to age-related penetrance, and that evaluation of the proband’s age should be considered for the assessment of this variant. Further evaluation of the patient’s clinical data revealed that she was the youngest patient in the cohort (16 years old at the time of data collection), and the INT^2^GRATE status was appropriately conservative in describing the variant in this patient. Patients 22 and 23 were positive for *VHL*:c.345C>A and *VHL*:c.532C>G, respectively, each presenting with one component tumor: a hemangioblastoma and a paraganglioma. The INT^2^GRATE assessment assigned code IV–II, indicating the combination of evidence in these patients was insufficient for a complete variant assessment. In these patients, the negative family history of VHL diagnosis suggests that the component tumor may be sporadic or constitutional with low penetrance.

In all patients with informative INT^2^GRATE variants (i.e., Positive or Negative), the clinical data was consistent with their INT^2^GRATE status, demonstrating the application of INT^2^GRATE in offering insights into the role of the germline *VHL* variants in relation to VHL syndrome in each patient (Supplementary Materials Tables S4–S6, Online INT^2^GRATE Portal).

### 3.5. INT^2^GRATE Variants and Clinical Actionability

After establishing the patient-specific applications, we investigated whether the INT^2^GRATE|VEF could also support variant actionability more globally. To achieve this, we evaluated the INT^2^GRATE variants and their related ACMG classifications and case reports in the literature. For the ACMG assessment, we evaluated the variant classifications provided by the reporting laboratory on patient reports in this study, in addition to those publicly available from all submitters in ClinVar.

The INT^2^GRATE Negative categories accurately identified the benign *VHL* variants and those associated only with ECYT2. All variants classified as benign (*n* = 15) and those with a pathogenic classification related to congenital erythrocytosis (*n* = 9) were INT^2^GRATE Negative (Figure 4C,D, Supplementary Materials Tables S5, S6 and S8). These variants are not clinically actionable in the context of VHL syndrome, and their INT^2^GRATE Negative status reflects this absence of clinical actionability.

Notably, 94% (75/80) of the variants reported as VUS were assessed to be INT^2^GRATE Negative (Figure 4C, Supplementary Materials Tables S5 and S6). In the ClinVar database, these variants were reported either as VUS due to insufficient clinical evidence or as likely benign by other submitters. This observation suggests that the INT^2^GRATE assessment is consistent with the B/LB submissions. Furthermore, the comprehensive VHL-specific clinical evidence from INT^2^GRATE can provide crucial clinical data for the variants labeled as VUS due to the absence of clinical information.

Among the P/LP germline *VHL* variants, 69% (20/29) were INT^2^GRATE Positive, consistent with the patients’ classic VHL syndrome and the actionability of these germline variants (Figure 4C). A remaining 21% (6/29) of the P/LP variants were INT^2^GRATE Negative. Four variants collectively in six individuals were *VHL*:c.292T>C (p.Tyr98His) in two unrelated individuals, *VHL*:c.562C>G (p.Leu188Val) in two unrelated individuals, *VHL*:c.388G>A (p.Val130Ile), and *VHL*: c.429C>T (p.Asp143=). We investigated these variants to determine whether the INT^2^GRATE Negative status was specific to the patient in our study or if the evidence supported their lack of actionability more broadly. Regarding *VHL*:c.292T>C (p.Tyr98His), in addition to two individuals with no VHL-related evidence in this study, we have previously reported this variant in an 82-year-old patient with urothelial cancer negative for all VHL-related evidence (personal and family features, tumors, and somatic variants) [8]. In the literature, this variant has been reported as a founder variant in the Black Forest region of Germany in individuals with pheochromocytoma [18,19,20]. Regarding *VHL*:c.562C>G (p.Leu188Val), in addition to two individuals with no VHL-related evidence in this study, we have previously reported this variant in two family members negative for all VHL-related evidence (personal and family features, tumors, and somatic variants) [8]. In the literature, this variant has been reported in the compound heterozygous state in congenital erythrocytosis and polycythemia [21,22,23,24]. The variants *VHL*: c.429C>T (p.Asp143=) and *VHL*:c.388G>A (p.Val130Ile) have also been reported in individuals with erythrocytosis [25,26] and not VHL syndrome.

### 3.6. INT^2^GRATE Variants and Distribution Along VHL Exons

**Figure 5:**
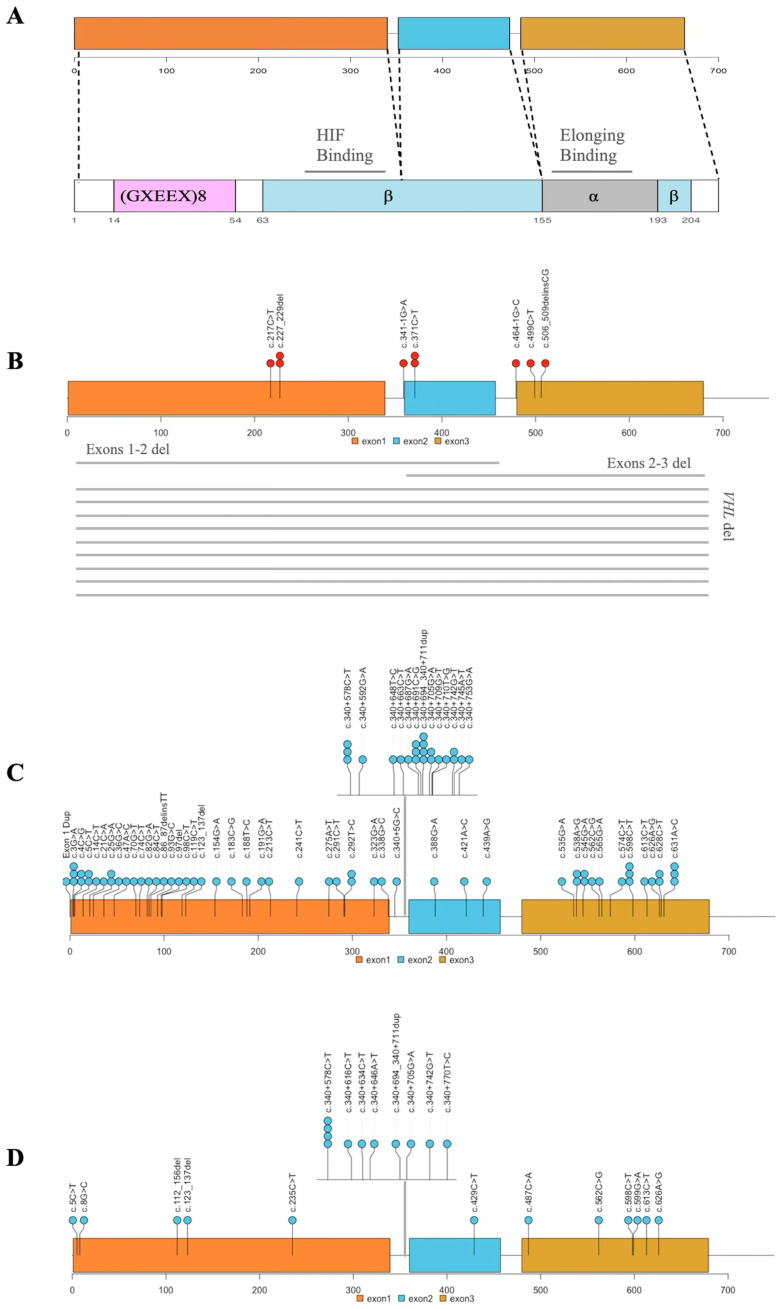
Distribution of the Informative INT^2^GRATE Variants Across the *VHL* Gene. (**A**) A schematic of the *VHL* gene is shown, including exons, the α- and β-structural domains, the HIF interaction domain, and the elongin C binding domain. Lollipop plots illustrate the distribution of variants across the gene: (**B**) the INT^2^GRATE Positive variants, (**C**) the INT^2^GRATE Negative variants in category III–II, and (**D**) the INT^2^GRATE Negative variants in category III–III. Variants located within the cryptic exon E1′, situated deep within intron 1, are shown in an inset within the intronic region in (**C**,**D**).

We assessed the distribution of the *VHL* variants across the three exons of the *VHL* gene to investigate their localization and to determine whether the variants from different INT^2^GRATE categories were confined to specific regions within the *VHL* (Figure 5). The majority of the INT^2^GRATE Positive variants (*n* = 11/20) were large deletions, either spanning multiple exons or the entire *VHL* gene, and null variants (c.341-1G>A, c.464-1G>C, c.506_509delinsCG, c.217C>T) (Figure 5A,B), Supplementary Materials Table S4). The number of missense variants was too small to provide insight into the potential enrichment of the loss-of-function missense variants in *VHL* in this study.

The INT^2^GRATE Negative variants were widely distributed. Several variants were categorized as III–II, and the same variants in other unrelated patients in this study fell in the III–II category (Figure 5C,D). Both INT^2^GRATE categories indicate the absence of VHL-specific evidence: patients with III–II variants displayed non-VHL tumors, and those with III–III variants had no tumors. While the variants in these overlapping INT^2^GRATE categories further signify that these *VHL* variants are not actionable, their distribution in both lollipop diagrams supports the lack of enrichment or the localization of INT^2^GRATE Negative variants in VHL regions.

### 3.7. Analysis of Somatic VHL Allelic Tumors

Hemangioblastomas and ccRCC are relatively common in VHL syndrome, but they are also frequently observed as isolated sporadic tumors in the absence of germline *VHL*. In such cases, although these tumors are not part of any constitutional syndrome, their mere presence in a patient with a germline *VHL* variant could lead to the incorrect attribution of the tumor to the germline VHL. The comprehensive INT^2^GRATE|VHL is designed to distinguish somatic differential tumors.

To better understand the molecular landscape of these sporadic tumors, we investigated somatic *VHL* alterations in 2539 patients with no germline alterations related to VHL syndrome. Of the *VHL* alterations, 154 were SNVs and 2394 were CNs, of which 1817 were deletions (either one copy or two copy deletions). Somatic profiles and tumor pathology evaluations indicated that, in most cases, these somatic *VHL* alterations were passenger alterations due to genomic instability, and not the driver events in the development of tumors associated with the *VHL* gene. Only 75 patients were diagnosed with ccRCC, and 6 had pancreatic neuroendocrine tumors, while 2 had paragangliomas. The paraganglioma tumors were SDH-deficient and, therefore, excluded from the list of VHL-related tumors (Supplementary Materials Table S9). Among the tumors with somatic *VHL* alterations, ccRCC was the most frequent sporadic *VHL*-associated tumor.

**Figure 6:**
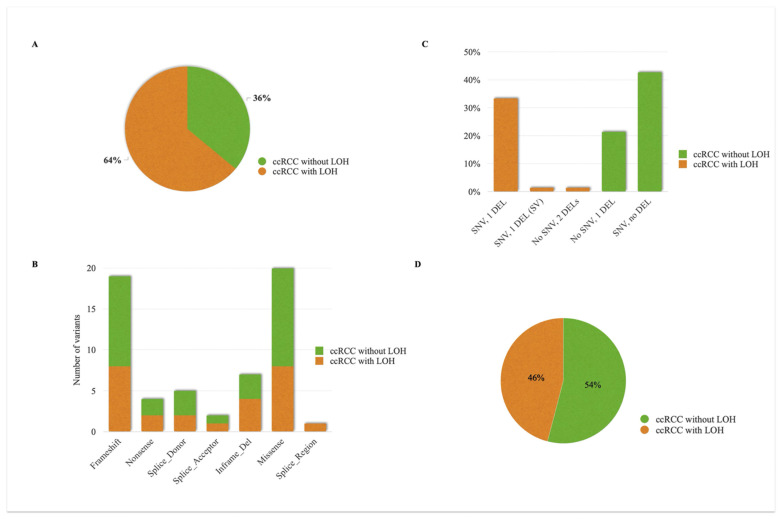
Molecular landscape of somatic *VHL* alterations in clear cell renal cell carcinoma (ccRCC) tumors. (**A**) Among patients with sporadic ccRCC, 64% exhibited LOH due to biallelic somatic inactivating alterations in *VHL*. (**B**) Different types of genetic alterations in somatic alleles are shown in the ccRCC cases with and without LOH, with the most common LOH mechanism involving one somatic SNV and one somatic copy deletion. (**C**) The types and frequency of somatic inactivating SNVs in the *VHL* showed no significant difference between the ccRCC cases with LOH and those without LOH. (**D**) Null *VHL* variants were slightly more frequent in the ccRCC cases without LOH compared to those with LOH. SNV: single nucleotide variant; DEL: copy number deletion of *VHL* allele; LOH: loss of heterozygosity.

Of the 75 patients with ccRCC, 36% (27/75) exhibited LOH due to biallelic somatic inactivating alterations in the *VHL* (Figure 6A). The most common LOH mechanism involved one somatic SNV and one somatic copy deletion in the VHL (Figure 6B). No significant differences were observed in the type of inactivating SNV (missense vs null) between the ccRCC cases with or without LOH (Figure 6C) or their distribution across the *VHL* exons (Supplementary Materials Figure S1). Null *VHL* variants (*n* = 37) were slightly more frequent in the ccRCC cases without LOH (54% or 17/37) compared to those with LOH (46% or 20/37) (Figure 6D). These results show that, in a proportion of the ccRCC cases, LOH and bilateral somatic *VHL* alterations may not be present. This could be attributed to assay limitations and the inability to detect possible complex or cryptic rearrangements in the *VHL*. Alternatively, alterations may not be present at the genomic level (e.g., epigenomic hypermethylation of the *VHL* promoter). This limitation in detecting LOH and somatic inactivating alterations in the *VHL* is addressed in the INT^2^GRATE|VEF to improve accuracy. The scenarios that require the somatic genetic entry in combination with other related evidence include I–II, I–III, II–I, II–II, II–III, and III–I (Supplementary Materials Table S1).

### 3.8. INT^2^GRATE|Variant Portal and Data Sharing

**Figure 7:**
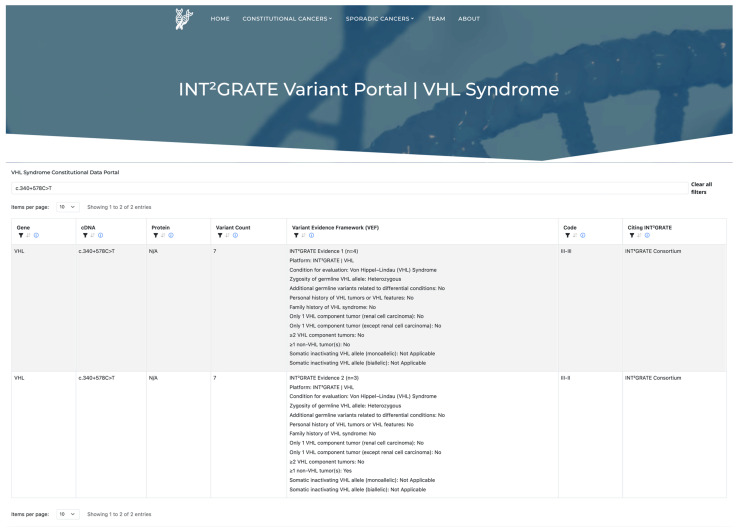
The INT^2^GRATE Variant Portal, publicly accessible at INT^2^GRATE.bwh.harvard.edu. A screenshot showing the search for *VHL*:c.340+578C>T. The variant is shown along with comprehensive details of associated clinical evidence. This variant was observed in seven unrelated patients (variant recurrence = 7). In four patients, the pattern of evidence was consistent with INT^2^GRATE Negative III–III (i.e., no tumor), and in three patients, it was consistent with INT^2^GRATE III–II (i.e., VHL tumor).

A primary goal of the INT^2^GRATE|Oncology Consortium is to advance the understanding of variant actionability in cancer through data sharing. To this end, we designed and launched the INT^2^GRATE Data Portal to publicly share the INT^2^GRATE variants and their associated evidence (INT^2^GRATEdata.bwh.harvard.edu). We programmatically assessed the frequency of each variant in the cohort, as well as the frequency of unique observations. A unique observation is defined when the same variant, even within the same INT^2^GRATE category, has a unique entry for a given parameter within the VEF framework (Figure 7). The portal facilitates the sharing of variants with detailed clinical information and frequency data, providing the scientific and clinical communities access to VHL-specific insights.

## 4. Discussion

An accurate assessment of the actionability of the germline variants is essential in cancer care. In VHL, clinical guidelines are largely based on clinical criteria, but establishing a molecular diagnosis requires the identification of a pathogenic or likely pathogenic germline *VHL* variant [9,15,16]. Similarly, clinical trials for targeted therapies in VHL disease, such as those involving hypoxia-inducible factor-2 alpha (HIF-2α) inhibitors or receptor tyrosine kinase (RTK) inhibitors, commonly have strict eligibility criteria based on genetic testing (ClinicalTrials.gov). Despite advances in molecular diagnostics, identifying the actionable germline variants remains challenging, as evidenced by the high prevalence of VUS among the reported variants. In VHL, these challenges are further compounded because tumors, such as RCC, that appear in hereditary VHL syndrome can also occur frequently as sporadic tumors without germline involvement. In fact, except for retinal hemangioblastomas, the majority of VHL component tumors are sporadic. As a result, clinicians may be inclined to overinterpret a *VHL* VUS in an individual with RCC or other component tumors, which could lead to over-screening of the patient and relatives. Furthermore, VHL tumors exhibit overlapping features with several differential genetic conditions (Supplementary Materials Table S2). Finally, the germline *VHL* variants could be genetically associated with the ECYT2 allelic disorder. The INT^2^GRATE|VHL addresses all these challenges through a comprehensive, evidence-based framework that incorporates constitutional, sporadic, differential, and allelic genetic conditions.

In developing the INT^2^GRATE|VEF, we incorporated a comprehensive set of evidence and parameters routinely used in clinical practice. We then crafted realistic scenarios based on actual clinical experiences and applied expert consensus to ensure conservative decision-making in each case. The VEF underwent review and finalization by the INT^2^GRATE’s multidisciplinary core committee, which includes board-certified medical geneticists specializing in cancer genetics and tumor profiling, clinicians and genetic counselors with expertise in hereditary cancer diagnostics, including VHL, and board-certified pathologists with expertise in molecular pathology and tumor profiling. The VEF parameters were shared with the INT^2^GRATE|Consortium members for data collection, and a digital version of the VEF was developed at the INT^2^GRATE Coordinating Center. All patient data were analyzed programmatically through the digital VEF to create the INT^2^GRATE Variants for analysis and sharing via the INT^2^GRATE Data Portal.

The underlying mechanism of VHL disease is loss of function, although the exact association between the genetic variants and the diverse VHL phenotypes is not yet fully understood. Recent research has demonstrated that splicing dysregulation in the E1’ and E2 regions of the VHL gene, leading to the formation of hypomorphic *VHL* variants, could be the underlying cause of erythrocytosis [25]. The E1’ region is a cryptic exon located deep in intron 1 of the *VHL* gene. Variants in this region create spliced isoforms containing the cryptic exon E1’, resulting in downregulation of VHL protein expression in patients with erythrocytosis. The INT^2^GRATE results were consistent with these findings, as all *VHL* variants across 33 individuals in the E1’ region were INT^2^GRATE Negative, showing the absence of all evidence related to VHL syndrome (Supplementary Materials Table S8). Furthermore, the synonymous *VHL*:c.429C>T (p.Asp143=) variant has been reported in homozygous or compound heterozygous states in erythrocytosis patients, and has been characterized to induce exon 2 skipping, resulting in decreased *VHL* expression [25]. The INT^2^GRATE Negative findings for the variants in E1’ and E2 aligned with their lack of association with VHL syndrome.

Consistent with the conservative INT^2^GRATE VEF, all 20 variants in the patients with classic VHL syndrome were INT^2^GRATE Positive, and all 24 benign variants or known variants associated with ECYT2 were INT^2^GRATE Negative. Interestingly, 94% (75/80) of the VUS variants were assessed to be INT^2^GRATE Negative. They were largely in the III–II category (*n* = 57), followed by the III–III category (*n* = 18), consistent with the absence of all comprehensive VHL-specific clinical evidence in these patients. Further investigation revealed that the variants in 20 individuals were located within the cryptic exon E1’ (deep intronic) in the *VHL* gene (as noted above; 33 variants were in E1’, 20 of which were VUS). The variants in this region are expressed at low levels in several human tissues, and have been associated with erythrocytosis [25]. The VUS classification in the remaining 60/80 cases is largely due to the absence of clinical information in the literature. Sharing these variants, along with their pertinent VHL-specific clinical evidence, on the INT^2^GRATE|Data Portal can provide critical support to clinical laboratories and expert groups in reclassifying these variants over time.

In our analysis, we identified six variants in four individuals as INT^2^GRATE negative [(*n* = 4, III–II; *n* = 2, III–III)], despite being reported as P/LP by the laboratory. We further examined these variants to determine whether the inconsistency was related to variations in VHL phenotypic expression among the individuals or a potential interpretation issue in assigning the P/LP classifications. Variants *VHL*:c.562C>G (p.Leu188Val), *VHL*: c.429C>T (p.Asp143=), and *VHL*:c.388G>A (p.Val130Ile) have been reported in association with erythrocytosis [21,22,23,24,25,26] and not VHL syndrome [8]. This observation demonstrates that the INT^2^GRATE program can efficiently differentiate between the involvement of variants in allelic disorders. The *VHL*:c.292T>C (p.Tyr98His) variant was present in two unrelated individuals (ages 81 and 83) with bladder and cervical cancer. We have previously reported this variant in an 82-year-old patient with urothelial cancer negative for all VHL-related evidence (personal and family features, tumors, and somatic variants) [8]. However, in the literature, this variant has been reported in association with pheochromocytoma and VHL Type 2C. The absence of all VHL-specific evidence in three of our patients, in contrast to the presence of pheochromocytoma in case reports, may suggest low penetrance of the variant or potential somatic allelic events causing sporadic pheochromocytoma. VHL tumors, other than retinal hemangioblastomas, are generally more common as sporadic tumors. VHL syndrome is estimated to account for 25% of CNS hemangioblastomas, less than 20% of pheochromocytomas, less than 10% of pancreatic neuroendocrine tumors, and 1% of RCCs [27,28,29]. A larger variant dataset could shed light on the full spectrum of VHL phenotypes in the individuals with these variants.

While VHL syndrome is typically considered an early-onset, high-penetrance condition, some variants may be associated with reduced penetrance. Clinical observations support age-related penetrance for component tumors in the individuals with VHL syndrome, with a mean age of diagnosis varying by tumor type: 22 years for endolymphatic sac tumors, 25 years for retinal hemangioblastoma, 30 years for pheochromocytoma, 30–32 years for CNS hemangioblastoma, 36 years for pancreatic lesions, and 39 years for renal cell carcinoma [30,31,32,33,34]. The risk for multiple tumors increases with age in people with VHL syndrome, and younger patients are less likely to be affected with one or more component tumors. We have considered age-related penetrance in the design of the VEF (INT^2^GRATE scenarios with codes II–V, II–VI, and II–VII), and variants from three patients fell in these categories (Supplementary Materials Table S7). One patient harbored *VHL*:c.208G>A, which was assigned code II–VI. This designation indicates that the VHL component tumors may be due to age-related penetrance and that the evaluation of the proband’s age should be considered in this variant assessment. The review of patient data confirmed this assessment, as the patient was 16 years old and had no VHL personal features yet, but her mother was diagnosed with VHL syndrome.

The INT^2^GRATE|VHL has several limitations. The platform analyzes the variants identified through DNA sequencing, but epigenetic alterations are not detectable by sequencing and, as such, are not evaluated. Studies have shown that methylation of the *VHL* promoter or consensus hypoxia-responsive element (HRE) can result in *VHL* loss, contributing to ccRCC progression [35,36,37]. Other limitations relate to somatic data. LOH at 3p is an early event in ccRCC, with 40–57% of alterations being somatic inactivating genetic alterations, and 10–15% resulting from epigenetic hypermethylation [38]. Our somatic *VHL* analysis showed that 36% of RCC tumors did not have LOH of the *VHL* locus. The LOH status in our study was based on genetic analysis and did not include copy-neutral LOH and methylation. Furthermore, cryptic alterations or complex rearrangements in the genome could not be assessed with standard testing and, therefore, were not investigated in this study. Another limitation is the lack of both somatic and germline sequencing data for every patient in this study. A subset of patients in Cohort 2 did not undergo constitutional cancer testing, and it is not clear whether the *VHL* alterations detected through tumor profiling—most of which were deletions—were truly somatic. Given that pathogenic germline *VHL* alterations, particularly deletions, are expected to be highly penetrant, and these patients did not have a syndromic tumor profile (i.e., had only one isolated component tumor), it is likely that these *VHL* alterations were somatic events. Nonetheless, the most accurate assessment requires a comprehensive analysis of both the germline and the somatic data for each individual. There are also limitations related to the sensitivity of the germline DNA testing. Studies have indicated that about 20% of the individuals with VHL syndrome have post-zygotic de novo alterations, and an estimated 5% of the individuals with VHL have somatic mosaicism [39]. Low-level mosaicism may not be detected or reported by the testing laboratories. All patients in this study have heterozygous *VHL* alterations, and therefore, the variants with low allelic fraction and the patients with no detectable germline *VHL* alterations were not included in this study.

It is noteworthy that INT^2^GRATE is primarily designed to evaluate the potential actionability of a given *VHL* variant in each patient rather than serving as a tool for the broad reclassification of variants. However, observing a consistent INT^2^GRATE pattern for a variant across multiple individuals over time could broaden its application in variant actionability. The INT^2^GRATE|Data Portal facilitates access to the variants and their VHL-related clinical evidence, as well as the frequency of variant recurrence. In this study, we identified several recurrent SNVs and indels with informative INT^2^GRATE categories. They include the INT^2^GRATE Negative variants (c.123_137del, c.25G>A, c.292T>C, c.340+705G>A, c.613C>T) in two unrelated patients; variants (c.340+691C>G, c.340+742G>T, c.3G>A, c.631A>C) in three unrelated patients; variants (c.340+694_340+711dup, c.545G>A, c.598C>T, c.5C>T, c.626A>G) in four unrelated patients; and c.340+578C>T in seven unrelated patients. Among the INT^2^GRATE Positive variants, c.227_229del and c.371C>T have been observed in two unrelated patients. As the variant database increases in size, the INT^2^GRATE|Data Portal will broaden its impact on variant actionability and may support the reclassification of variants. The portal facilitates the sharing of variants with detailed clinical information and frequency data, providing the scientific and clinical community access to VHL-specific insights.

## 5. Conclusions

In conclusion, the INT^2^GRATE|VHL presents a novel and comprehensive evidence-based framework that integrates germline, somatic, tumor, and associated clinical data, making them publicly accessible via the INT^2^GRATE variant portal. The INT^2^GRATE analysis of the *VHL* variants is an effective approach for determining the actionability of the constitutional *VHL* variants while excluding sporadic, differential, and allelic genetic conditions. The INT^2^GRATE Data Portal provides scientists, clinicians, and laboratories with unparalleled access to comprehensive variants and their associated clinical data. Through data sharing, the INT^2^GRATE Oncology Consortium is committed to advancing the clinical actionability assessment of genomic variants, fostering informed decision-making, and advancing precision oncology.

## Figures and Tables

**Table 1:** Patient Cohorts in the INT^2^GRATE|VHL Program.

Patient Cohorts Based on Availability of Data Type	Numbers
Total patients in the study	2672
Breakdown of Cohort 1	
Patients had somatic *VHL* variants and detailed tumor data, evaluated in genetics, and had germline *VHL* variants.	11
Patients had no somatic sequencing data, had detailed tumor data, were evaluated in genetics, and had germline *VHL* variants.	122
Breakdown of Cohort 2	
Patients had somatic *VHL* variants and detailed tumor data, were evaluated in genetics, and had no germline *VHL* variants.	638
Patients had somatic *VHL* variants and detailed tumor data, were not evaluated in genetics for any hereditary cancer, and had no germline sequencing data *.	1901

* Germline VHL status extrapolated from somatic sequence data and clinical data.

**Table 2:** The demographic of patients in the INT^2^GRATE|VHL program who were positive for the germline *VHL* variant.

Sex and Age Distribution of Patients with the Germline VHL Variant	Numbers (%)
Female	98 (74%)
Male	35 (26%)
Median Age	54
Patient with VHL Component Tumor	27 (20%)
Patient with no VHL Component Tumor	106 (80%)
Patient with No Tumor or Cancer Diagnosis	24 (18%)
Patient with Positive family History of VHL Diagnosis	17 (13%)
Patient with Negative Family History of VHL Diagnosis	114 (86%)
Patient with Unavailable Family History of VHL Diagnosis	2 (2%)

**Table 3:** Distribution and prevalence of VHL tumors in patients with germline *VHL* variant in the INT^2^GRATE|VHL program.

VHL Tumor Type	VHL Tumors (%)	Females (%)	Males (%)	Mean Age	Patients with Family History of VHL Diagnosis
**Hemangioblastomas**					
CNS hemangioblastoma	16 (12%)	15 (12%)	1 (1%)	36.9	11 (9%)
Retinal hemangioblastoma	11 (9%)	9 (7%)	2 (2%)	32.3	8 (6%)
**Renal lesions**					
Multiple renal cysts	13 (10%)	11 (9%)	2 (2%)	32.9	10 (8%)
Renal cell carcinoma	9 (7%)	4 (3%)	5 (4%)	44.3	5 (4%)
**Pheochromocytoma**	4 (3%)	2 (2%)	2 (2%)	30.5	3 (2%)
**Paraganglioma**	3 (2%)	2 (2%)	1 (1%)	31	1 (1%)
**Pancreatic lesions**					
Pancreatic cysts	14 (11%)	11 (9%)	3 (2%)	30.6	10
Neuroendocrine tumors of the pancreas	1 (1%)	1 (1%)	0	54	0
**Endolymphatic sac tumors**	1 (1%)	1 (1%)	0	30	0
**Epididymal and broad ligament cystadenomas**	0	0	0	0	0

## Data Availability

The original contributions presented in this study are included in the article, the public INT^2^GRATE Portal, and the Supplementary Materials. Further inquiries can be directed to the corresponding author.

## Supplementary Materials

The following supporting information can be downloaded at: https://www.mdpi.com/article/10.3390/cancers17132173/s1, Figure S1: Distribution of somatic SNVs across *VHL* exons in clear cell RCC (ccRCC tumors, with and without loss of heterozygosity (LOH); Table S1: INT^2^GRATE Variant Evidence Framework (VEF) for VHL Syndrome; Table S2: VHL-differential conditions and associated genes in the INT^2^GRATE | Variant Evidence Framework; Table S3: Distribution and prevalence of non-VHL tumors in patients with germline *VHL* variant in the INT^2^GRATE | VHL Program; Table S4: INT²GRATE Positive variants in the *VHL* gene; Table S5: INT^2^GRATE Negative III-II variants in the *VHL* gene; Table S6: INT^2^GRATE Negative III-III variants in the *VHL* gene; Table S7: Germline *VHL* variants in the INT^2^GRATE Neutral category; Table S8: Germline *VHL* variants in the cryptic exon E1’ and associated Iin the INT^2^GRATE category; Table S9: Somatic *VHL* allelic tumors in the INT^2^GRATE study.
