# Supplementary figures and images for: Advancing the Landscape of Clinical Actionability in Von Hippel–Lindau Syndrome: An Evidence-Based Framework from the INT2GRATE Oncology Consortium
Source: Cancers (Basel). 2025 Jun 27;17(13):2173. doi: 10.3390/cancers17132173 (PMC12248699; doi:10.3390/cancers17132173)

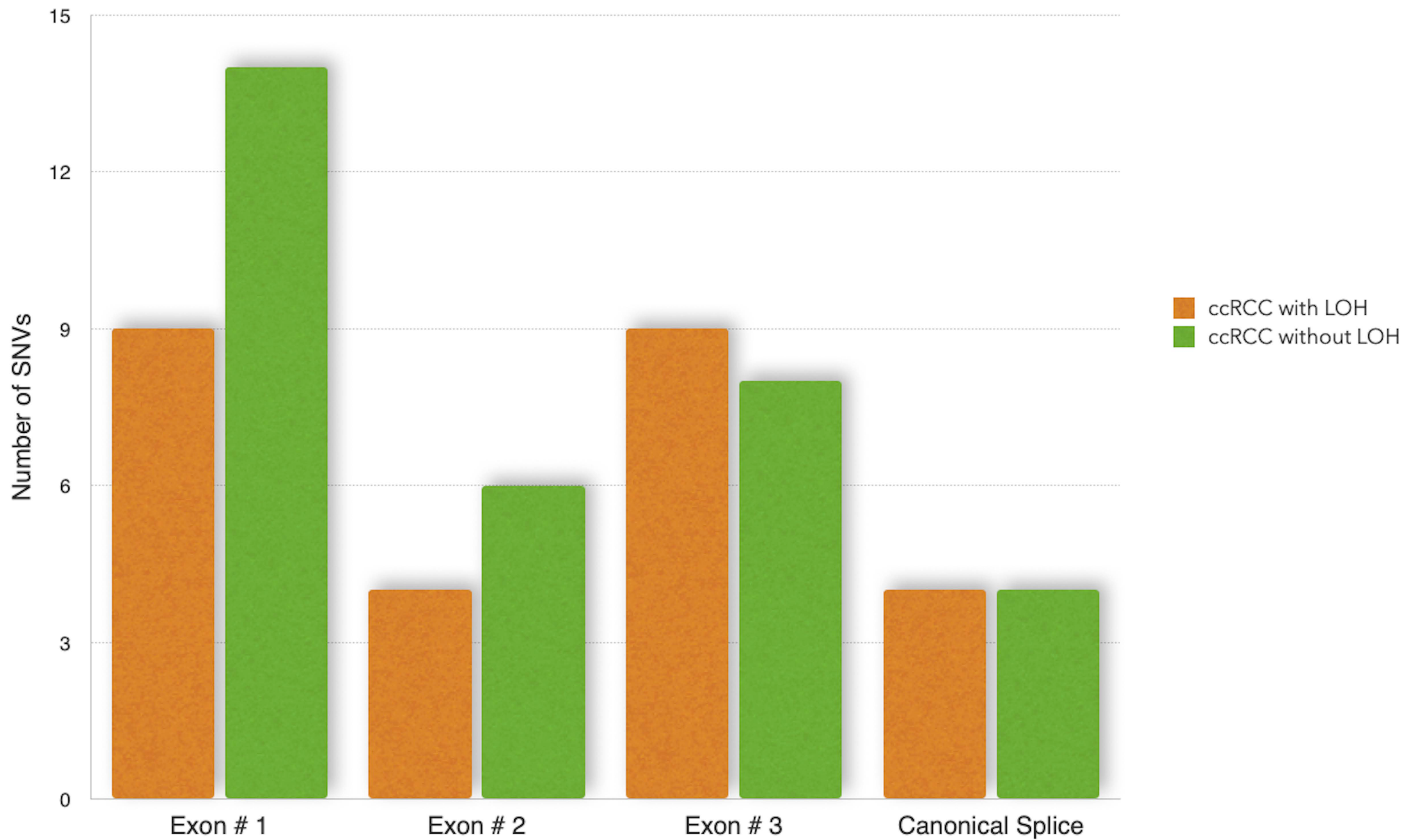

Supplement: Supplementary file 1 [file cancers-17-02173-s001.zip › Supplementary Figure S1_INT2GRATE VHL Manuscript_FINAL_May062025.pdf]
